# Ejaculated Mouse Sperm Enter Cumulus-Oocyte Complexes More Efficiently In Vitro than Epididymal Sperm

**DOI:** 10.1371/journal.pone.0127753

**Published:** 2015-05-21

**Authors:** Honggang Li, Pei-hsuan Hung, Susan S. Suarez

**Affiliations:** 1 Family Planning Research Institute, Tongji Medical College, Huazhong University of Science and Technology, Wuhan, China; 2 Wuhan Tongji Reproductive Medicine Hospital, Wuhan, China; 3 Department of Biomedical Sciences, College of Veterinary Medicine, Cornell University, Ithaca, New York, United States of America; Universidad Nacional Autónoma de México, MEXICO

## Abstract

The mouse is an established and popular animal model for studying reproductive biology. Epididymal mouse sperm, which lack exposure to secretions of male accessory glands and do not precisely represent ejaculated sperm for the study of sperm functions, have been almost exclusively used in studies. We compared ejaculated and epididymal sperm in an in vitro fertilization setting to examine whether ejaculated sperm enter cumulus-oocyte complexes more efficiently. In order to prepare sperm for fertilization, they were incubated under capacitating conditions. At the outset of incubation, ejaculated sperm stuck to the glass surfaces of slides and the incidences of sticking decreased with time; whereas, very few epididymal sperm stuck to glass at any time point, indicating differences in surface charge. At the end of the capacitating incubation, when sperm were added to cumulus-oocyte complexes, the form of flagellar movement differed dramatically; specifically, ejaculated sperm predominantly exhibited increased bending on one side of the flagellum (a process termed pro-hook hyperactivation), while epididymal sperm equally exhibited increased bending on one or the other side of the flagellum (pro-hook or anti-hook hyperactivation). This indicates that accessory sex gland secretions might have modified Ca^2+^ signaling activities in sperm, because the two forms of hyperactivation are reported to be triggered by different Ca^2+^ signaling patterns. Lastly, over time, more ejaculated than epididymal sperm entered the cumulus oocyte complexes. We concluded that modification of sperm by male accessory gland secretions affects the behavior of ejaculated sperm, possibly providing them with an advantage over epididymal sperm for reaching the eggs in vivo.

## Introduction

The mouse is an established and popular animal model for studying mechanisms that regulate sperm movement and fertilization in mammals. Epididymal mouse sperm have been almost exclusively used instead of ejaculated sperm for in vitro experiments, because collecting ejaculated mouse sperm is much more difficult and expensive than obtaining epididymal sperm. Whereas, semen of various mammalian species can be collected by artificial vagina and/or electroejaculation, artificial vaginas are not available for collecting mouse semen and mouse sperm collected by electroejaculation have low viability and poor rates of in vitro fertilization [1]. In addition, mouse semen collected by electroejaculation coagulates abnormally [2]. Therefore, mouse ejaculated sperm have been collected in the uterus of a female via precise timed mating [3, 4], which is more costly and time consuming.

Although epididymal sperm morphologically resemble ejaculated sperm, there are differences between them. During the process of ejaculation, epididymal sperm are mixed with secretions of male accessory sex glands and the cytoplasmic droplet is shed from sperm. Proteins secreted by the accessory sex glands affect sperm in various ways, one way being that some proteins interact with proteins or lipids on the sperm membranes to inhibit premature sperm capacitation. For example, it has been reported that seminal vesicle secretion 2 (SVS2) binds to ganglioside GM1 in the postacrosomal region of mouse sperm to inhibit epididymal sperm capacitation in vitro [5, 6]. Also, serine protease inhibitor kazal-type-like protein (SPINKL) from mouse seminal vesicles is found to delay the capacitation of epididymal mouse sperm in vitro [7]. In view of these and other effects of accessory gland secretions, one could conclude that using epididymal mouse sperm to study sperm function does not precisely represent how sperm function in vivo. Thus, this study was undertaken to test the hypothesis that ejaculated and epididymal sperm behave differently.

## Materials and Methods

### Animals

Adult male (13–17 weeks old) and female (11–15 weeks old) CD1 mice used in this study were purchased from Charles River Laboratories International Inc. (Wilmington, MA). Animals were housed in a room with 12 h light per day. For sample collection, mice were euthanized by carbon dioxide asphyxiation followed by cervical dislocation. All animal procedures were approved by the Institutional Animal Care and Use Committee at Cornell University (Protocol Number: 2009–0011).

### Sperm Collection and Capacitation

A mouse sperm capacitating medium [8] was used for sperm preparation and capacitation.

To obtain ejaculated sperm, female mice were injected IP with 10 IU PMSG (Calbiochem, Billerica, MA) followed by 10 IU hCG (Calbiochem, Billerica, MA) 48 h later for superovulation. Mating was timed to occur 12–14 h after hCG injection, by introducing a female into the cage of a singly housed male. The observation of mating was conducted under a red light. Time of ejaculation was recorded when the male suddenly and briefly became very still while grasping the female [9], and mating was verified by detection of a vaginal plug. Sperm were recovered from the female no later than 30 min after coitus. The uterus was dissected and then rinsed with sperm medium. The cervical end of the uterus was raised to avoid leakage of semen during these procedures. A cut was made near the uterotubal junction of each uterine horn and 200 μl medium were gently pipetted into each uterine horn at the cervical end to flush out sperm into a 1.5 ml eppendorf tube. COCs were also collected from the oviducts of the mated female, as described below.

At the time of coitus, a different male of the same age as the mated male, and which had mated 2–7 days earlier, was euthanized. Epididymal sperm were collected as described previously [10]. Briefly, caudal epididymides were removed, cleaned, and then placed under mineral oil in a 35 mm Petri dish. Several cuts were made in the coiled tubules near the vas deferens, and the emerging thick fluid was gently pulled out with fine tweezers and moved under oil into a droplet of mouse medium in the dish. Sperm were allowed to disperse for 10 min in the incubator before sperm numbers were determined by using a hemocytometer.

Concentrations of ejaculated and epididymal sperm were adjusted to 10 x 10^6^/ml with medium and sperm were incubated at 37°C under 5% CO_2_ in humidified air to stimulate capacitation.

**Fig 1:**
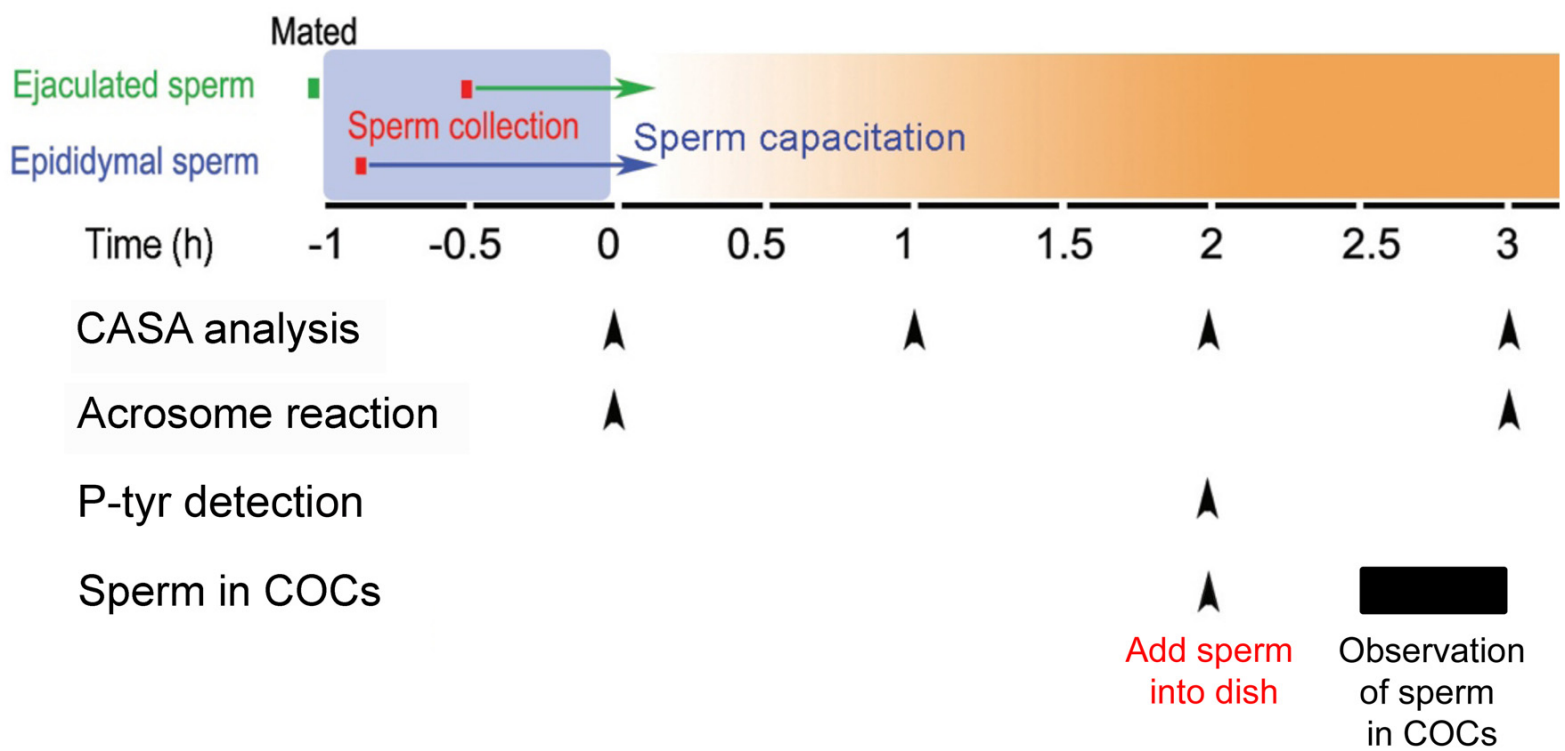
Diagram of experimental design and timeline of sample collection. After mating of one male was observed, another male was sacrificed within 10 min to collect epididymal sperm. Ejaculated and epididymal sperm were adjusted to 10 x 10^6^/ml and capacitated for up to 3 h. CASA analysis, acrosome status, and sperm penetration of COCs were performed at the time points indicated by arrowheads. Numbers of ejaculated and epididymal sperm were evaluated 30 to 60 min after sperm were added to the COCs.

A detailed experimental time line is illustrated in Fig 1.

### Recording of Sperm Movement

Slides and coverslips used for analysis of sperm motility were coated with 0.5% (w/v) agarose (Fisher Scientific, Rockford, IL, Cat number BP 1356–100) in purified water and dried overnight. At 0, 1, 2, and 3 h after incubation under capacitating conditions, 10 μl of sperm suspension were taken from each sample (10 x 10^6^/ml), placed on a slide, and covered with a 22 mm^2^ coverslip to create a chamber with approximately 20 μm deep. Samples were recorded at 60 frames/sec with a JVC SR-S365U videocassette recorder (JVC, Long Beach, CA), using a 10x negative phase contrast objective (Carl Zeiss Microscopy, LLC, Thornwood, NY) and a Dage CCD 72 video camera (Dage-MTI, Inc., Michigan City, IN). At least 20 microscope fields were recorded for analysis of each sample.

### Sperm Motility and Computer-assisted Semen Analysis

Percentages of motile sperm were determined by visual analysis of the videotapes and motility was analyzed using an IVOS CASA system (Hamilton Thorne Inc., Beverly, MA). Instrument settings were as follows: frame rate, 60 Hz; frames acquired, 30; minimum contrast, 40; minimum cell size, 12 pixels; static VAP cutoff, 10 μm/s; static VSL cutoff, 5 μm/s; progressive VAP threshold, 10.1 μm/s. For each sample, at least 200 sperm were evaluated. Average path velocity (VAP), straight-line velocity (VSL), curvilinear velocity (VCL), amplitude of lateral head displacement (ALH), beat cross frequency (BCF), straightness (STR), and linearity (LIN) were evaluated.

### Assessment of Acrosomal Status

Percentages of sperm with intact acrosomes were determined using Coomassie blue to stain acrosomes, as previously described [10]. At least 200 sperm were evaluated in each sample.

### Preparation of Cumulus Oocyte Complexes (COCs)

**Fig 2:**
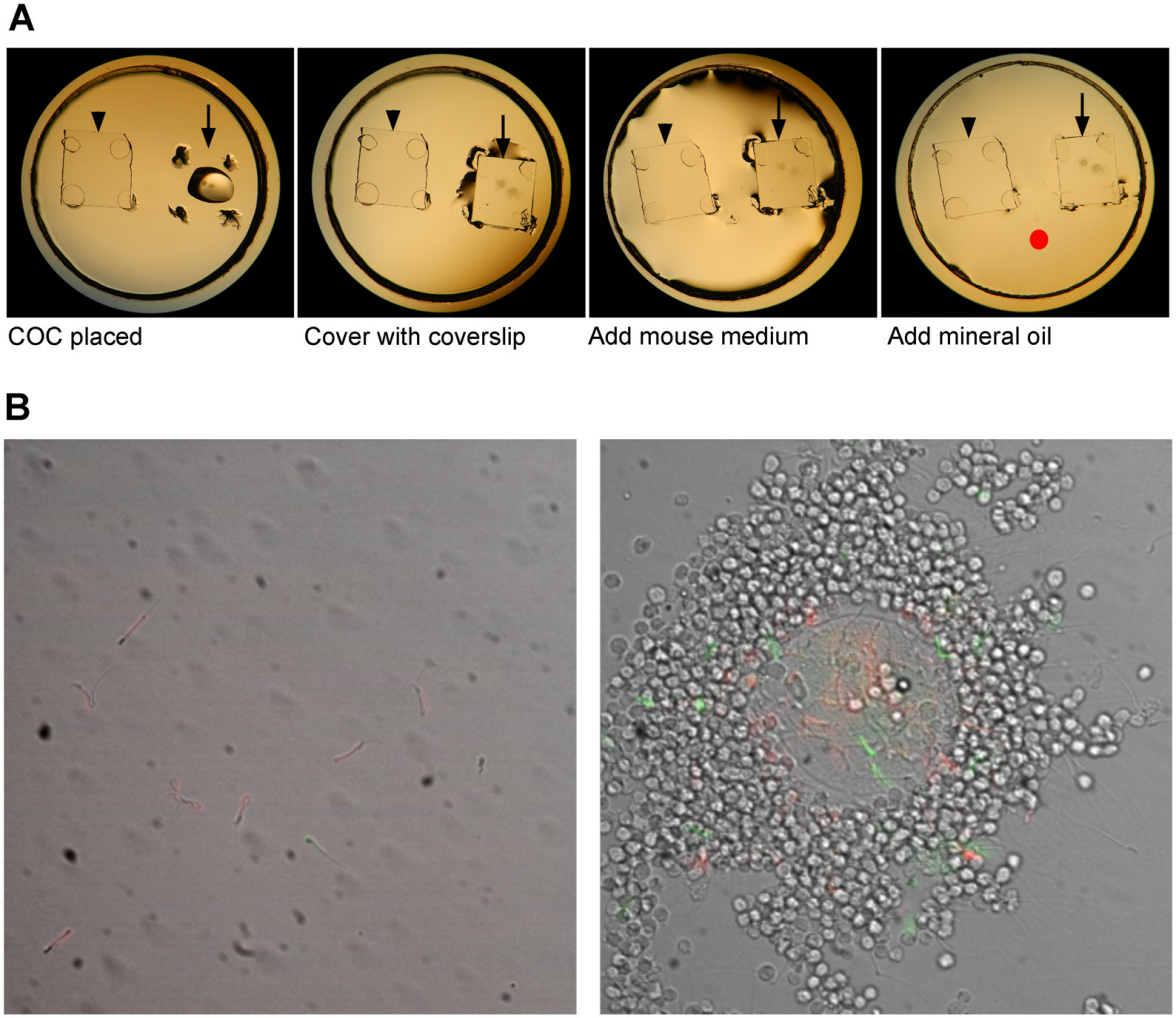
Setup for analysis of sperm penetration of COCs. A. A COC (arrow) was placed on the 14 mm diameter recessed glass insert of a glass-bottomed 35-mm petri dish and covered with a 3 mm x 3 mm coverslip supported by four dabs of silicon grease. As a negative control, four dabs of silicon grease supporting a coverslip were placed in the same recessed insert (arrowhead). A total of 50 μl mouse medium was added into the insert and then covered with mineral oil. Sperm were added at the red dot. B. Examples of MitoTracker labeled ejaculated and epididymal sperm in the negative control coverslip (left) and in the COCs (right). Fluorescence is difficult to detect in some sperm in Fig 2B, because they were located above or below the focal plane; however, the focal plane was moved up and down during video imaging in order to clearly detect all sperm.

COCs were obtained from the mated females used for ejaculated sperm collection. Both oviducts were removed and placed in 500 μl medium in a petri dish right before dissecting the uterus of the mated female for sperm recovery. The large cumulus mass was recovered from each oviduct by tearing open the wall of oviduct with a 27g needle. The cumulus mass was gently separated into COCs containing 2–4 oocytes each. A COC was placed on the 14 mm diameter recessed glass insert of a 35 mm petri dish (MatTek Corporation, Ashland, MA; Part Number: P35G-0-14-C) and covered with a 3 mm x 3 mm coverslip supported by four dabs of silicon grease (Corning Inc., Corning, NY) (Fig 2A, arrow). As a negative control, four dabs of silicon grease supporting a coverslip were placed in the same recessed insert (Fig 2A, arrowhead). A total of 50 μl medium was added into the insert and then covered with mineral oil. The observation dish was incubated at 37°C under 5% CO_2_ in humidified air for at least 1.5 h before addition of sperm.

### Sperm Staining and Analysis of Cumulus Penetration

After incubation under capacitating conditions for 1 h, epididymal sperm and ejaculated sperm were alternatively stained with 25 nM MitoTracker Red CMXRos or 5 nM MitoTracker Green FM (Molecular Probes, Eugene, OR) for 15 min. After 3 washes in the mouse medium at 600 x g for 3 min, sperm were incubated at 37°C for at least 15 min in a 1.5 ml eppendorf tube before insemination. At 2 h after the initiation of capacitation, a 10 μl aliquot each of motile stained ejaculated and epididymal sperm was taken from the upper layer of each tube and the aliquots were mixed together. Then, 10 μl of the mixture (50 x 10^3^ sperm) were added into the center of the glass insert at equal distance to the two coverslip preparations in the petri dish (Fig 2A, red dot). After 30–60 min of incubation, the numbers of ejaculated and epididymal sperm in the COC were determined using epifluorescence optics coupled with dim transmitted light through a 40x objective (Fig 2B, right). To count the sperm under the coverslip lacking COCs, the observation dish was microwaved for 5 sec to immobilize the sperm (Fig 2B, left). Numbers of red and green sperm were determined using a dual band excitation filter set. Images were taken with a Sensicam em High Performance camera (PCO-TECH Inc., former “Cooke Corporation”, Romulus, MI) controlled by IPLab 4.0 software (BD Biosciences, Rockville, MD) (Fig 2B). The ratios of ejaculated/epididymal sperm in the COCs were standardized to the ratios of ejaculated/epididymal sperm in the COC-free preparation in the same dish.

### SDS-PAGE and Western Blot

Sample preparation for SDS-PAGE was as previously described [11]. Briefly, sperm were washed in 1 ml of phosphate-buffered saline (PBS) at 600 x g for 3 min. Sperm proteins were extracted with SDS sample buffer [12], boiled for 5 min, and centrifuged at 10,000 x g for 3 min to remove insoluble particles. The supernatants were collected and stored at -20°C. Before gel electrophoresis, samples were thawed and a final concentration of 5% β-mercaptoethanol was added [13]. After boiling and centrifugation, protein extracts equivalent to 2 x 10^6^ sperm were resolved in 8% polyacrylamide gels and transferred onto PVDF membranes (Life Technologies, Grand Island, NY). To detect protein tyrosine phosphorylation, membranes were blocked with Tris-buffered saline containing 0.1% (v/v) Tween-20 (TBST) containing 5% gelatin (Bio-Rad Laboratories, Hercules, CA) for 2 h at room temperature and then probed with protein phosphotyrosine antibody at 200 ng/ml (4G10, Millipore Co., Temecula, CA) [11] overnight at 4°C. Membranes were washed in TBST 3 times for 10 min and then incubated in goat-anti-mouse IgG conjugated with horseradish peroxidase (1:8,000 dilution) (Santa Cruz Biotechnology Inc., Dallas, TX) for 1 h at room temperature. Membranes were washed in TBST 3 times for 10 min, and reactive proteins were visualized by enhanced chemiluminescence (Thermo Scientific, Rockford, IL) using FluorChem E System (ProteinSimple, San Jose, CA). To detect β-tubulin (as a loading control), membranes were stripped with buffer containing 2% SDS, 100 mM β-mercaptoethanol, and 62.5 mM Tris-HCl (pH 6.8) for 10 min at 55°C, blocked in 5% skim milk, and incubated with β-tubulin antibody (1:1,000 dilution; Accurate Chemical & Scientific Corporation, Westbury, NY) overnight at 4°C then incubated in goat-anti-mouse IgG conjugated with horseradish peroxidase (50 ng/ml) (Jackson ImmunoResearch Laboratories, Inc., West Grove, PA) for 1 h at room temperature.

### Statistical Analysis

Paired *t*-tests were used when comparing data from ejaculated and epididymal sperm from the same male. One sample t-tests were performed when comparing the adjusted ratio of ejaculated sperm/epididymal sperm in the COCs. Percentage data of acrosome-intact sperm, percentage of sticky sperm, and percentage of motile sperm were transformed using arcsine (square root) before statistical analyses were performed. A *P* value of less than 0.05 was chosen to indicate statistical significance. Data are presented as mean ± standard deviation (SD).

## Results

### Ejaculated sperm exhibited stickiness to glass surfaces

First, we tested the hypothesis that ejaculated and epididymal mouse sperm move differently. This hypothesis was based on the possibility that the presence of the cytoplasmic droplet on the flagellum of epididymal sperm and the modification of the plasma membranes of ejaculated sperm by accessory gland secretions could affect movement speeds or patterns. Video recordings of swimming behaviors of sperm on coverslipped glass slides, taken shortly after collection, revealed that most ejaculated sperm adhered continuously or intermittently to the glass surfaces by their heads (74.8 ± 11.8%, N = 3), despite the presence of BSA in the medium, which is known to prevent sperm from sticking to glass [14](S1 Movie). After incubation under capacitating conditions for 2 h, the numbers of strongly adherent ejaculated sperm were reduced (40.9 ± 4.5%, N = 3). Sperm that broke free of the glass were hyperactivated and often repeatedly adhered briefly to the glass (S2 Movie). In contrast, a lower percentage of epididymal sperm adhered to glass surfaces (6.9 ± 6.3%, N = 3, *P* = 0.002 when compared to ejaculated sperm)(S3 Movie), either by their heads or by the cytoplasmic droplets, suggesting that accessory gland secretions modified surface properties of sperm.

To prevent sperm from sticking to the glass in subsequent experiments, we coated all slides and coverslips with 0.5% agarose.

### Epididymal sperm and ejaculated sperm moved differently in vitro

**Fig 3:**
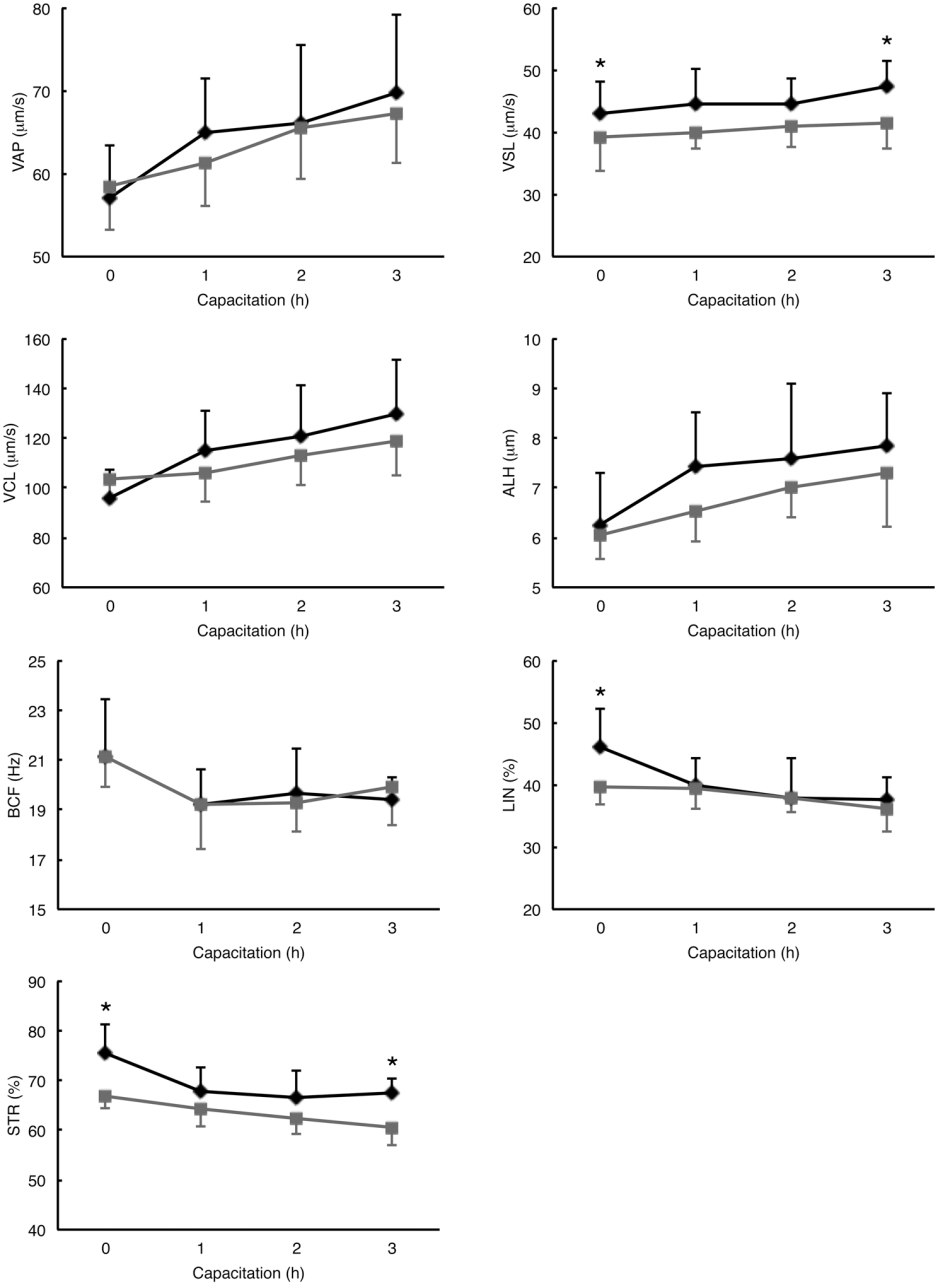
CASA analysis of ejaculated (diamond) and epididymal (rectangle) mouse sperm. Sperm samples were collected every hour during capacitation. Data are presented as mean ± SD (N = 5). Asterisks indicate significant difference (*P* < 0.05) between ejaculated and epididymal sperm, determined by paired t-test.

To eliminate the contribution of individual animal to variation, we compared ejaculated and epididymal sperm from the same male collected on different days (ejaculated sperm and then the epididymal sperm 2–7 days later, N = 5). At the initiation of incubation under capacitating conditions, more ejaculated sperm were motile than epididymal sperm (80.4 ± 7.0% and 69.6 ± 6.0% for ejaculated and epididymal sperm, respectively; *P* = 0.035, N = 5). In samples taken at the beginning of incubation under capacitating conditions, ejaculated sperm had higher VSL, STR, and LIN than epididymal sperm (Fig 3). At 3 h of incubation under capacitating conditions, the ejaculated sperm still had higher VSL and STR than the epididymal sperm (Fig 3), even though about 60% of the epididymal sperm had lost the cytoplasmic droplet by that time.

Previously, we reported that epididymal mouse sperm exhibit two distinct hyperactive swimming patterns, pro-hook and anti-hook, which are triggered by two different Ca^2+^ signaling pathways [10]. In pro-hook hyperactivation, the large dominant flagellar bend is oriented in the same direction as the hook of the sperm head, whereas, in anti-hook hyperactivation, the dominant flagellar bend forms in the direction opposite to that of the hook of the sperm head. At 2 h after the initiation of incubation under capacitating conditions, more than 90% of ejaculated sperm displayed pro-hook hyperactivation (S4 Movie), although some sperm briefly switched to anti-hook hyperactivation. In contrast, the swimming patterns of epididymal sperm were heterogeneous, with approximately 50% showing pro-hook and 50% showing anti-hook hyperactivation (S5 Movie). Most epididymal sperm also repeatedly switched between the two bending patterns, but they remained swimming in each pattern roughly the same length of time. Both hyperactivation bending patterns were consistently observed in epididymal sperm throughout the time of incubation.

### Rates of spontaneous acrosome reactions were similar in epididymal and ejaculated sperm

At the initiation of incubation, the percentage of acrosome-intact sperm from the ejaculated sperm was higher than that in the epididymal sperm (98.4 ± 1.2% and 95.4 ± 1.0% for ejaculated and epididymal sperm, respectively; *P* = 0.019, N = 5). At 3 h of incubation, there was no significant difference in the percentages of acrosome-intact sperm between the ejaculated sperm (76.0 ± 10.3%) and epididymal sperm (79.1 ± 5.7%).

### More ejaculated sperm entered cumulus oocyte complexes (COCs)

Red and green MitoTracker dyes were used to distinguish ejaculated sperm and epididymal sperm and to reveal them moving within the cumulus matrix. While MitoTracker Green labels all sperm (live or dead), MitoTracker Red CMXRos only labels live sperm. This did not interfere with our observation as only sperm from the upper layer (motile sperm) of each tube were taken, and only motile sperm can penetrate the cumulus. Furthermore, in order to minimize possible effects of dyes, we switched the dyes used to distinguish sperm in each replicate experiment.

More sperm (both ejaculated and epididymal sperm) were found under the coverslips containing COCs than under the coverslips lacking COCs. Because of the differences in the percentage of motile ejaculated and epididymal sperm at the beginning of incubation, silicon grease spots supporting a coverslip were placed in the same recessed insert as the cumulus masses. When the ratios of ejaculated/epididymal sperm in the COCs were standardized to the ratios of ejaculated/epididymal sperm in control preparations that lacked COCs, it was determined that more ejaculated sperm entered the COCs than epididymal sperm in one hour (Table 1, *P* = 0.026), indicating that modification of sperm during ejaculation provides an advantage to sperm for entering COCs.

**Table 1:** Ejaculated sperm enter COCs more rapidly than epididymal sperm.

Experiments	COCs	Control	Adjusted Ratio
**1**	41/64 (0.64)	16/35 (0.46)	1.40
**2**	111/160 (0.69)	48/100 (0.48)	1.45
**3**	47/28 (1.68)	33/38 (0.87)	1.93
**4**	70/46 (1.52)	74/67 (1.10)	1.38

Data are presented as ejaculated/epididymal sperm (ratio). The adjusted ratio is the ratios of ejaculated/epididymal sperm in the COC standardized to the ratios of ejaculated/epididymal sperm in the COC-free control preparation in the same dish.

### Ejaculated sperm showed higher level of protein tyrosine phosphorylation under capacitating conditions

**Fig 4:**
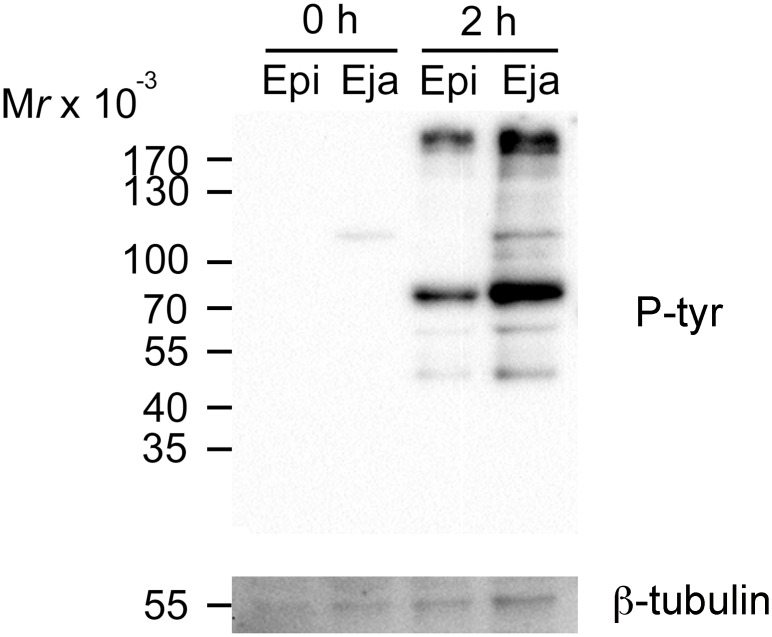
Western blot analysis of sperm protein tyrosine phosphorylation. Ejaculated and epididymal sperm samples were collected at the beginning (0 h) and after 2 h of incubation under capacitating conditions. Protein lysates equivalent to 2 x 10^6^ sperm were loaded per lane and resolved with an 8% polyacrylamide gel for protein tyrosine phosphorylation detection (p-tyr). β-tubulin was also probed as a loading control.

To examine whether ejaculated and epididymal sperm responded differently to capacitating conditions, we collected sperm samples at the initiation of capacitation and at 2 h of incubation (the time sperm were added to the COCs) for protein tyrosine phosphorylation analysis. At the beginning of incubation (0 h), ejaculated sperm showed a weak single band of tyrosine-phosphorylated protein between 110 and 130 kDa, while epididymal sperm did not show any visible band (Fig 4). At 2 h incubation under capacitating conditions, both ejaculated and epididymal sperm extracts showed significant increases of protein tyrosine phosphorylation, indicating that both had undergone capacitation to some extent. Nevertheless, more tyrosine-phosphorylated protein bands with higher signal intensity were detected in extracts of ejaculated sperm, suggesting that ejaculated sperm had undergone a higher degree of capacitation than epididymal sperm at the time they were introduced to the COCs.

## Discussion

Our observations show that ejaculated sperm swim differently than epididymal sperm and are more efficient at penetrating the cumulus oophorous. These results indicate that exposure to accessory sex gland secretions provide sperm with an advantage for fertilizing eggs in vivo, where oocytes are surrounded by cumulus at the site and time of fertilization in the mouse [9].

Proteins in seminal plasma have been reported to coat sperm during ejaculation and play roles in fertilization in vivo. As discussed above, accessory gland proteins in mice, such as SVS2 and SPINKL play important roles in regulating sperm capacitation [5–7]. A recent study showed that when seminal vesicles were surgically removed from males (SVX males) and the males were then mated with females, the pregnancy rate was only 35% of that produced by the control males [15], indicating that seminal plasma enhances the success of fertilization. In addition, embryos flushed from females mated with the SVX males had reduced cleavage rate [15]. Another study showed that the fertility of SVS2 deficient mice was severely reduced, as most sperm died in the uterus and only very few sperm reached the oviductal ampulla, where fertilization takes place [16]. Our study further suggests that reduced efficiency of sperm penetration of cumulus, could have also contributed to the lower rate of 2-cell stage embryos resulting from mating with SVS2-deficient or SVX males [15].

For our study, ejaculated sperm were collected from uteri shortly after mating. This method has been established and reported by other groups [3, 4]. During the procedure, sperm were very briefly exposed to uterine secretions and were quickly diluted by sperm medium. In the rat and hamster, ejaculated sperm are transported rapidly *en masse* through the cervix into the uterus, where their motility is activated within a few minutes [17]. Nevertheless, activation of motility can also be seen after dilution of vaginal sperm in Tyrode’s solution; therefore, it can be achieved without uterine-specific secretions [17]. Bicarbonate is the likely candidate for the observed effect, because it has been shown to activate epididymal mouse sperm [18] and secretion of bicarbonate into the uterus by endometrial epithelial cells is high during estrus in the mouse [19].

At 2 h of incubation, the time when aliquots of sperm were introduced to the COCs, we observed that the swimming patterns of ejaculated and epididymal sperm differed. Previously, we reported that epididymal mouse sperm exhibit two distinct patterns of hyperactivation, pro-hook and anti-hook, which are triggered by different Ca^2+^ signaling pathways [10]. Pro-hook hyperactivation can be induced by treating uncapacitated epididymal mouse sperm with 4-aminopyridine (4-AP) or procaine [10] to activate CATSPER channels in the flagellum and trigger an influx of extracellular Ca^2+^ in the principal piece of the flagellum. Anti-hook hyperactivation can be induced by treating mouse sperm with thimerosal, which is thought to raise cytoplasmic Ca^2+^ by releasing it from intracellular stores, particularly those found in the redundant nuclear envelope at the base of the sperm flagellum [10, 20]. In the present study, ejaculated sperm predominantly developed pro-hook hyperactivation during incubation in capacitating medium in vitro, while epididymal sperm switched back and forth between pro-hook and anti-hook hyperactivation. These differences indicate that the dynamics of the two pathways that are thought to raise cytoplasmic Ca^2+^ in sperm differ between epididymal and ejaculated sperm. It is possible that the sustained pro-hook hyperactivation observed in ejaculated sperm was promoted by components in seminal plasma that inhibit release of Ca^2+^ from intracellular stores. This possibility is supported by the observation that anti-hook hyperactivation dominates when uncapacitated epididymal mouse sperm are treated by a combination of 4-AP and thimerosal and also when capacitated epididymal sperm are treated with thimerosal [6]. Alternatively, components of seminal plasma could somehow increase activation of CATSPER channels to the point at which the effect of Ca^2+^ influx in the principal piece dominates over Ca^2+^ release from intracellular stores. Further investigation is needed to determine whether the distinct flagellar bending patterns between capacitated epididymal and ejaculated sperm are due to a different balance of mechanisms of that increase cytoplasmic Ca^2+^ levels.

The presence of cytoplasmic droplets could have caused a shift in Ca^2+^ signaling dynamics toward dominance of the Ca^2+^ store release pathway; however, we observed that epididymal sperm with cytoplasmic droplets were able to display pro-hook flagellar bending, suggesting that the cytoplasmic droplet does not play a role in determining sperm swimming patterns (S6 Movie).

Contrary to the previous finding that epididymal sperm predominantly displayed pro-hook hyperactivation during incubation under capacitating conditions [10], we detected both pro-hook and anti-hook bending equally in epididymal sperm after 2 h of incubation in capacitating medium (S5 Movie). This difference might be attributed to the use of different mouse strains in the two studies. In the current study, CD1 mice were used, while F1 hybrid cross of C57BL/6J3BALB/cByJ males were used in the previous work [10]. It is intriguing to consider that differences in hyperactivation patterns could provide sperm from one strain with advantages over those of another strain.

We found more sperm (both ejaculated and epididymal) under the coverslip that covered the COCs than under the control coverslip (Table 1). This may have been because the cumulus matrix slowed down sperm movement and trapped sperm [21]. Another possibility is that sperm were attracted to the COCs by chemotaxis. It has been proposed that, like sea urchin sperm, mammalian sperm exhibit the ability to detect chemical cues that guide them to the egg (reviewed by [22, 23]); however, there is no direct evidence that sperm were guided to the COCs in our experiments by chemotaxis.

In our study, ejaculated sperm showed a higher level of protein tyrosine phosphorylation than epididymal sperm at 2 h of incubation (Fig 4). Other researchers also found that ejaculated sperm were more capacitated than epididymal sperm by analyzing acrosomal exocytosis [4]. Interestingly, these results contradict studies showing that SVS2 and SPINKL delay mouse epididymal sperm capacitation in vitro [5–7]. Diluting seminal plasma while collecting ejaculated sperm from the uterus might somehow accelerate the removal of decapacitation factors from the sperm surface and hence potentiate sperm capacitation; however, more studies are needed to elucidate the mechanism responsible for differential capacitation.

Mouse ejaculated and epididymal sperm protein tyrosine phosphorylation had not been compared side by side until this study. The hexokinase p95/116 has been found to be tyrosine-phosphorylated in uncapacitated mouse epididymal sperm [24]. It is interesting that we detected a tyrosine phosphorylated band between 110 and 130 kDa, presumably the p95/116 hexokinase, in ejaculated sperm but not in epididymal sperm (Fig 4). We suspect that the hexokinase p95/116 signal in ejaculated sperm is much stronger than that in epididymal sperm, therefore the p95/116 hexokinase signal in epididymal did not get picked up due to the short exposure time to accommodate the strong signal in ejaculated sperm. Further investigation is needed to confirm our findings and to reveal the biological meanings of p95/116 in sperm function.

It is also interesting to note that ejaculated sperm exhibited stickiness to the glass surfaces, while epididymal sperm did not. This indicates a difference in cell surface charge, which may reflect the coating of ejaculated sperm by accessory gland secretions; however, further investigation is needed to identify the molecules in accessory sex gland secretions responsible for the stickiness of ejaculated mouse sperm to glass surfaces and whether this change in surface property plays a role in sperm migration in the female tract. In cattle, binder of sperm proteins (BSP) secreted by the seminal vesicles coat the plasma membrane of the sperm head and midpiece by interacting with membrane phospholipids [25–27]. The BSP coat enhances sperm binding to the epithelial lining of the oviduct, which leads to formation of a sperm storage reservoir [28].

In conclusion, we demonstrated that ejaculated mouse sperm enter COCs more efficiently than epididymal mouse sperm. This could be the result of changes in the surface coat of sperm, which might have accounted for stickiness to glass, and/or changes in Ca^2+^ signaling that regulate hyperactivation patterns. It is becoming increasingly clear that accessory gland secretions modify sperm behavior. These modifications may provide sperm with advantages for reaching and fertilizing oocytes in vivo.

## Supporting Information

S1 Movie Mouse ejaculated sperm sticking to glass. Most ejaculated sperm adhered continuously or intermittently to the glass surfaces by their heads, despite the presence of BSA in the medium.
(M4V)

S2 Movie Hyperactivated ejaculated sperm breaking free of glass. After incubation under capacitating conditions for 2 h, fewer ejaculated sperm adhered strongly to the glass. Sperm that broke free were hyperactivated and often repeatedly adhered briefly to the glass.
(M4V)

S3 Movie Mouse epididymal sperm moving freely on slide. Unlike ejaculated sperm that adhered to glass via their heads, epididymal sperm swam freely.
(M4V)

S4 Movie Ejaculated sperm displaying pro-hook hyperactivation. At 2 h after the initiation of incubation under capacitating conditions, more than 90% of ejaculated sperm displayed pro-hook hyperactivation.
(M4V)

S5 Movie Swimming patterns of hyperactivated epididymal sperm. At 2 h after the initiation of incubation under capacitating conditions, epididymal sperm showed heterogeneous swimming patterns, with approximately 50% showing pro-hook and 50% showing anti-hook hyperactivation. Most epididymal sperm also repeatedly switched between the two bending patterns, but they remained swimming in each pattern roughly the same length of time.
(M4V)

S6 Movie Pro-hook flagellar bending in epididymal sperm with cytoplasmic droplets. Hyperactivated epididymal sperm with cytoplasmic droplets displayed pro-hook flagellar bending.
(M4V)
